# The Impact of Nurse Managers' Transformational Leadership on Nurses' Work Engagement: A Cross-Sectional Study

**DOI:** 10.1155/jonm/9980485

**Published:** 2025-06-16

**Authors:** Anu Nurmeksela, Katju Schavoronkoff, Krista Jokiniemi

**Affiliations:** ^1^Department of Nursing Science, University of Eastern Finland, Kuopio, Finland; ^2^Helsinki University Hospital, Helsinki, Finland

**Keywords:** nurse, nurse manager, transformational leadership, well-being, work engagement

## Abstract

This study aimed to describe nurses' evaluations of their work engagement, their perceptions of their managers' transformational leadership, and the relationships between these factors. A cross-sectional study design was employed. The Transformational Leadership Scale was used to measure the transformational leadership qualities of nurse managers. The Utrecht Work Engagement Scale (UWES-9) was used to measure nurses' work engagement. The data were analyzed using statistical tests, the Pearson correlation coefficient, and linear regression analysis. Among the work engagement subscales, nurses scored highest on dedication to work (mean 4.14, SD 1.22) and lowest on vigor (mean 3.68, SD 1.9). For transformational leadership subscales, giving feedback and rewards received the lowest scores (mean of 2.64, SD 1.02), while management of the nursing process scored the highest (mean 3.25, SD 0.92). The correlation between transformational leadership and work engagement was statistically significant (*r* = 0.367, *p* < 0.001). In conclusion, transformational leadership by nurse managers may increase nurses' work engagement. Nurse managers can develop and incorporate transformational leadership traits through training programs. Further research is needed to understand the mechanisms linking transformational leadership to nurses' work engagement.

## 1. Introduction

Addressing the nursing shortage requires various measures, including the implementation of effective leadership, the enhancement of employee well-being, and the improvement of working conditions [1]. Healthy and content employees represent an employer's greatest asset, as they are highly productive and deeply dedicated to the organization. Factors that positively affect well-being at work include inspiring and motivating leadership, the work community atmosphere, and the professional competence of the leaders and staff [2, 3]. Previous research has indicated that high levels of work engagement are positively associated with improved well-being [4]. Employees with strong work ability are more likely to experience positive work-related well-being. Furthermore, the relationship between work engagement and work ability has been shown to be mediated by job satisfaction [5]. Ensuring well-being at work is a collective responsibility of the entire work community. However, leaders are crucial in optimizing the processes that facilitate this well-being. Workplace well-being is a key factor in an individual's overall personal welfare [1].

Previous studies have found that over half of the nurses have experienced work burnout [6, 7]. Even though the experience of moral distress has been moderate, the intensity has been identified as high [8]. This is highly concerning, especially when considering the COVID-19 pandemic, which has further strained the healthcare system and reduced nurses' work engagement [9], increased their work burnout [10–12], and undermined their well-being. Nurse leaders need tools to retain nurses in their roles. One of the key responsibilities of nurse leaders, now and in the future, is to ensure and enhance nurses' well-being at work. Transformational leadership plays a crucial role in enhancing work engagement [13, 14] and the overall well-being of nurses [15, 16]. This leadership style focuses on relational competencies such as effective communication, emotional intelligence, and fostering professional growth [17–21]. By prioritizing these aspects, transformational leaders create a positive and supportive work environment where nurses feel valued and motivated [22]. Research has shown that transformational leadership is associated with increased job satisfaction, reduced burnout, and higher levels of work engagement among nurses. It also promotes resilience, organizational commitment, and lower stress levels [23–28]. In essence, transformational leadership not only improves the work atmosphere but also ensures that nurses are better equipped to provide excellent patient care. This approach is vital for retaining skilled nurses and maintaining a healthy, motivated workforce nurses' well-being at work.

## 2. Background

The literature describes various concepts related to transformational leadership and work engagement. Work engagement has often been studied as a consequence of work well-being. However, work well-being is a multifaceted concept that can be examined from various perspectives. It includes positive dimensions, such as work engagement and job satisfaction, as well as negative dimensions, such as work burnout, workaholism, and stress [29]. This study examines the positive dimensions of well-being at work through the lens of work engagement, recognizing it as an outcome of positive well-being experiences within the work environment.

Schaufeli and Bakker [30] initially defined work engagement as a genuinely positive state of emotion and motivation at work. Work engagement encompasses the positive dimensions of well-being experienced in the workplace. Three key concepts of work engagement are vigor, dedication to work, and absorption in work tasks [30]. Vigor is characterized by energy, the desire to invest in work, persistence, and willingness to try even in the face of difficulties. Dedication manifests as an experience of the meaning of work, where the employee expresses pride, challenge, enthusiasm, and inspiration. Both desire and dedication embody the positive opposites of exhaustion and cynicism. Absorption is characterized by a deep state of concentration and commitment to work, where the employee experiences enjoyment, time passes unnoticed, and detaching from work may feel challenging [29–33]. Work engagement increases when the demands of work and available resources are balanced, whereas stress and work burnout increase as work demands become excessive. Work engagement reduces work burnout and enhances job satisfaction, whereas workaholism and stress elevate the risk of work burnout [3, 29].

Transformational leadership encompasses characteristics that closely align with the principles of engagement leadership [34]. Transformational leadership encourages, inspires, and motivates employees to perform in ways that create meaningful change [35]. Four key concepts of transformational leadership are influence (charisma), inspirational motivation, intellectual stimulation, and individualized consideration [35]. Transformational leaders inspire and motivate their team by establishing a compelling vision and encouraging its achievement. This leadership approach fosters a sense of purpose and enthusiasm among nurses, thereby promoting higher levels of work engagement [34, 36]. Transformational leadership is further characterized by strong interaction, evident in the provision of feedback and rewards. These leaders are attentive to the individual needs and professional development of their team members [35]. By offering personalized support and mentorship, they help nurses feel valued and understood, which in turn enhances engagement. Moreover, transformational leaders engage with employees on a personal level, hold regular meetings with staff, and delegate responsibilities [35]. In addition, they engage staff in their work by encouraging them to overcome challenges and exceed expectations. A fundamental notion in transformational leadership is that leaders inspire their staff through their actions, creating a motivating environment [35]. Transformational leaders empower nurses by fostering job autonomy and shared decision-making [17], which serve as intrinsic motivation sources and enhance nurses' engagement in their work [37]. The characteristics of transformational leadership can be incorporated into one's leadership approach and developed through training and practice [38]. Research has shown that transformational leadership is particularly well-suited to today's constantly changing world, where leaders need to engage in innovative thinking that can swiftly respond to upcoming changes [39, 40].

Several previous studies have shown the positive influence of nurse leaders' relational leadership styles, such as transformational leadership, on nursing, patient, and organizational outcomes [17, 19, 27, 28, 41, 42]. Nurse managers' transformational leadership style has been observed to influence nurses' lower stress levels [17, 27, 28] and overall health [17, 42]. It also contributes to a healthy work environment [28, 42], empowerment [15, 17, 42], and autonomy [17, 42], all of which enhance job satisfaction [17, 42]. The relationships have been both direct and indirect, with transformational leadership acting as a moderator or mediating factor. In Finland, transformational leadership has been examined in relation to various nursing outcomes, including patient care quality [43, 44], empowerment [19], and medication safety [45]. However, to the best of our knowledge, there remains an empirical research gap concerning the relationship between transformational leadership and work engagement within the Finnish nursing context.

Transformational leadership and work engagement can be explored through the Job Demands–Resources (JD-R) theory [46], which explains how the organizational environment affects employee well-being and performance [47, 48]. According to the JD-R theory, key aspects include job demands and job resources. Job demands include aspects of a job that require sustained effort and are associated with costs, such as high workload and conflicting demands. Job resources help achieve work goals, reduce job demands, and promote personal growth, such as social support and job autonomy. Job demands can lead to a health impairment process resulting in burnout, while job resources lead to a motivational process resulting in increased work engagement, characterized by vigor, dedication, and absorption [29, 33]. Job demands and resources interact, with resources helping to manage high demands. Transformational leaders inspire and motivate nurses by appealing to higher ideals and values. This can foster a sense of purpose and commitment, leading to increased dedication and vigor. By empowering nurses and providing support, transformational leaders create an environment where nurses feel valued and capable. This empowerment can enhance their absorption in work tasks. Transformational leaders ensure that nurses are equipped with the necessary resources to effectively manage job demands. This support helps nurses maintain high levels of engagement and performance. This study aimed to describe nurses' assessments of their work engagement, their managers' transformational leadership, and the relationships between these concepts. Reporting is following the Reporting of Observational Studies in Epidemiology (STROBE) checklist [49].

In this study, the following research questions are addressed:

  RQ1. How do nurses assess their work engagement?
  RQ2. How do nurses assess their nurse managers' transformational leadership?
  RQ3. Is there a relationship between transformational leadership and work engagement?”

## 3. Materials and Methods

### 3.1. Study Design

This was a cross-sectional, descriptive study.

### 3.2. Data Collection and Sampling

The study targeted all specialized care nursing staff (*N* = 858) from a central hospital in eastern Finland. A convenience sampling method was employed, and data were collected through an electronic questionnaire administered in February 2022. The organization's contact person contacted the target group through an email with a link to the questionnaire. The questionnaire was distributed to all nursing professional groups, including registered nurses (defined in the Finnish context to encompass public health nurses, midwives, and paramedics) as well as practical nurses, across six operational units: acute and emergency care services, surgical operations, inpatient wards, outpatient clinics, women's and children's diseases, and mental health and substance abuse services. Nurse managers and leaders were excluded from the study. In this study, the term “nurse” is used to refer to all nursing staff. The survey remained open for a month, and after 2 weeks, nurses received a reminder to participate in the study.

### 3.3. Measures

The questionnaire consists of two pre-established measures: The Transformational Leadership Scale (TLS) [50] and the Utrecht Work Engagement Scale (UWES-9) [30, 31].

The Finnish TLS was developed to create a reliable and culturally sensitive instrument [51]. The development process included a literature review, evaluations, and comments by an expert panel, as well as a pilot study [50]. Since then, the TLS instrument has been validated in several studies, with Cronbach's alpha values ranging from 0.91 to 0.97 [50], 0.88 to 0.94 [52], and 0.878 to 0.968 [45]. The original TLS comprises a total of 64 questions categorized into three sections: background questions (10 items), nurse managers' leadership practices (43 items), and the nursing director's leadership practices (11 items). This study utilized background questions and those pertaining to nurse managers' leadership practices. Background questions contain demographic questions and an evaluation of the quality of the work unit's operations. The nurse managers' leadership practice section includes four subscales: ethical leadership (14 items), management of the nursing processes (16 items), giving feedback and rewards (6 items), and support for professional development (7 items). Nursing staff were asked to evaluate their managers using a five-point Likert scale (1 = fully disagree, 2 = partially disagree, 3 = I do not know, 4 = partially agree, and 5 = fully agree) [50]. Previous research has indicated that transformational leadership is considered excellent when assessed with a mean score greater than 4 [44].

The UWES-9 scale consists of nine questions divided into three subscales: vigor (3 items), dedication (3 items), and absorption (3 items). The statements are presented on a seven-point Likert scale (0 = never, 1 = almost never, 2 = rarely, 3 = sometimes, 4 = often, 5 = very often, and 6 = always) [30]. In the evaluation and discussion of the results, the level of work engagement based on the research data from the Finnish Institute of Occupational Health (*N* = 16,335) was utilized [32]: very low < 1.5, low 1.5–3.5, moderate 3.51–4.5, high 4.51–5.5, very high > 5.5.

### 3.4. Data Analysis

The data were analyzed with SPSS for Windows 27.0 (IBM Corporation, Armonk, NY). The respondents' background variables are described using descriptive statistics, including frequencies, percentages, means, and standard deviations (Table 1). The quality of the work unit's operations was assessed on a scale of 0–10. Quality ratings were categorized into three groups: 0–6, 7–8, and 9–10. The data were analyzed using the one-way analysis of variance (ANOVA), the Mann–Whitney *U* test, the Kruskal–Wallis test, the Pearson correlation coefficient, and linear regression analysis. The distributions of the sum variables were examined separately within each grouping variable using the Kolmogorov–Smirnov test. If the test result was *p* < 0.05, nonparametric tests were employed; otherwise, parametric tests were applied. In cases where statistically significant differences were identified among groups in comparisons involving more than two groups, further analysis of the group means was conducted using the Bonferroni post hoc test [53]. The transformational leadership and work engagement subscales were analyzed using nonparametric methods (Mann–Whitney *U*-test or Kruskal–Wallis test) for mean comparisons. The relationship between transformational leadership style and work engagement was analyzed using the Pearson correlation coefficient and linear regression analysis. A correlation coefficient between 0.50 and 1.00 indicates a strong relationship, between 0.30 and 0.50 indicates a moderate relationship, and between 0 and 0.30 indicates a weak relationship. The threshold for statistical significance was set at *p* < 0.05 [53].

### 3.5. Ethical Considerations

The research process adhered to good scientific practice in all phases. Research approval was obtained from a research organization (blinded for review). The study was reported accurately, honestly, and openly in accordance with the established ethical principles [54]. Participation in the study was voluntary, and the data were collected and analyzed anonymously. Participants were provided with an information sheet about the study before taking part in the survey. Informed consent was obtained at the beginning of the survey and participants could withdraw from the study at any time. Permission to use the TLS was requested from, and granted by, Professor Tarja Kvist. The UWES-9 scale is freely available for noncommercial research and educational purposes [33].

## 4. Results

### 4.1. Background Variables

A total of 154 nurses responded to the questionnaire, yielding a response rate of 18%. The majority of participants (83.8%) were registered nurses, while 9% were paramedics. Most respondents were female (88.2%) with an average age of approximately 42 years, ranging from 22 to 65. Their work experience ranged from less than 1 year to over 30 years, averaging 16 years. Experience in their current unit ranged from less than 1 year to over 25 years, with an average of around 8 years. A significant portion (44.1%) had worked in their current unit for less than 5 years. In addition, most respondents (82.6%) reported having a permanent employment relationship and working in shifts (76.2%).

The largest group of respondents (38.7%) worked in the acute care and emergency unit. Operational units employed 22%, inpatient wards 14%, outpatient clinics 14.7%, women and children's competence centers 6%, and mental health and substance abuse services 4.7%.

### 4.2. Nurses' Work Engagement

Nurses assessed their overall work engagement with a mean score of 3.87 (SD 1.12). They evaluated work engagement across three subscales: vigor (mean 3.68, SD 1.19), dedication (mean 4.14, SD 1.22), and absorption (mean 3.80, SD 1.12). Commitment to work was rated the highest, while vigor received the lowest rating (Table 1).

The quality of the work unit's operations was assessed to have a mean score of 7.36. Approximately 22% of respondents evaluated the work unit's quality as 0–6, just over half (51%) rated it 7–8, and slightly less than a third (27%) rated it 9–10 (Table 1).

Nurses' assessments of the quality of the working unit's operations showed statistically significant differences across various dimensions of work engagement. Higher ratings of work engagement correlated with higher ratings of unit operation quality (*p* < 0.001). Nurses who rated the unit's work quality in the 0–6 range experienced lower overall work engagement (mean 3.02, SD 1.15) compared to those who rated it in the 7–8 range (mean 3.82, SD 1.18) or 9–10 (mean 4.73, SD 0.73). Differences among all these groups were statistically significant (*p*=0.00–0.007) (Table 1).

The same phenomenon is observed across all aspects of work engagement (*p* < 0.001). Nurses who rated the quality of work unit operations as 9–10 experienced the highest levels of vigor (mean 4.59, SD 0.70), while those who rated it 0–6 experienced the lowest levels of vigor (mean 2.73, SD 1.19). Similarly, nurses who rated the quality of work unit operations as 0–6 (mean 3.09, SD 1.15) and 7–8 (mean 3.73, SD 1.07) experienced the lowest levels of absorption, whereas those who rated it 9–10 experienced the highest levels of absorption (mean 4.60, SD 0.82). Statistically significant differences were observed among all groups across different subscales of work engagement (*p* 0.001–0.027) (Table 1).

### 4.3. Nurses' Assessments of Nurse Managers' Transformational Leadership

Nurses assessed their managers' transformational leadership with a mean score of 3.15 (SD 0.99). However, providing feedback and rewards was rated lower (mean 2.64, SD 1.02) compared to ethical leadership (mean 3.23, SD 1.15), management of the nursing processes (mean 3.25, SD 0.92), and supporting professional development (mean 3.17, SD 1.11) (Table 1).

Nurses who had worked in their current unit for 5–10 years assessed their nurse managers' transformational leadership as less well-implemented (mean 2.74, SD 1.05) compared to nurses who had worked in their current unit for less than 5 years (mean 3.45, SD 0.89). The difference between these groups was statistically significant (*p* < 0.001) (Table 1).

The higher nurses assessed the quality of the work unit, the higher they assessed their managers' transformational leadership (*p* < 0.001). Those nurses who assessed the unit's work quality as between 0 and 6 perceived the lowest level of nurse manager transformational leadership (mean 2.64, SD 0.86), and those who rated it between 9 and 10 perceived the highest level of nurse managers' transformational leadership (mean 3.54, SD 1.03). There was a statistically significant difference between the two lowest categories (*p*=0.036) and the lowest and highest categories (*p* < 0.001) (Table 2).

### 4.4. Relationships Between Transformational Leadership and Nurses' Work Engagement

A scatterplot showed that the relationship between transformational leadership and work engagement was positive and linear and did not reveal any bivariate outliers. The correlation between transformational leadership and work engagement was statistically significant (*r* = 0.367, *p* < 0.001). The strongest correlation was found between transformational leadership and vigor (*r* = 0.391, *p* < 0.001). Transformational leadership and dedication were moderately correlated (*r* = 0.352, *p* < 0.001). A weak positive correlation was observed between transformational leadership and absorption (*r* = 0.293, *p* < 0.001) (Table 2).

Transformational leadership was statistically significantly associated with vigor, dedication, and absorption (*p* < 0.001). The *R*^2^ values for the equations were 0.153, 0.124, and 0.086; that is, 15.3% of the variance in vigor was predictable from the level of transformational leadership, 12.4% of the variance in dedication was predictable from the level of transformational leadership, and 8.6% of the variance in absorption was predictable from the level of transformational leadership (Table 3).

### 4.5. Reliability of Instruments

The reliability of the instruments was excellent, as Cronbach's values were 0.87–0.94 for the UWES-9 and 0.92–0.97 for TLS.

## 5. Discussion

This study aimed to describe nurses' assessments of their work engagement and their nurse managers' transformational leadership and to examine the relationship between transformational nursing leadership and nurses' work engagement. Nurses experienced work engagement at a moderate level. Among the dimensions of work engagement, nurses felt the most dedication to their work and the least vigor. Overall, nurses rated the implementation of their nurse managers' transformational leadership positively, though feedback and rewards received the lowest ratings across all aspects.

According to the JD-R theory, reinforcing job resources, such as social support and job autonomy, fosters personal growth, facilitates the achievement of work goals, and helps mitigate job demands [30]. In this study, the aspects of transformational leadership (ethical leadership, management of the nursing processes, giving feedback and rewards, and support for professional development) are included in these resources.

In this study, nurses rated the management of nursing processes highest among the subareas of transformational leadership, followed by ethical leadership and support for professional development. Currently, society and healthcare organizations are undergoing continuous transformation due to the aging population, the global nursing shortage, rapid technological development, and economic challenges [1, 55]. These challenges also affect nursing management and nurse managers, as they are on the frontline. Nurse managers hold a key position in overseeing nursing processes, and their effectiveness in this role directly influences nurses' work performance and outcomes [56, 57]. Transformational leaders can create an open and safe work environment by encouraging staff to participate in decision-making [17, 22, 37]. Nurse managers may encounter ethical challenges, particularly when operating under economic and productivity pressures [56, 57]. Nursing staff need to experience ethical leadership, as it ultimately influences patients' perceptions and experiences of care. A transformational leader values the professional knowledge and skills of nurses [35], thereby fostering independent decision-making [37]. Today, nursing staff value managers who recognize their expertise and support their professional growth and career development, factors that are also important for enhancing nurses' work engagement [58–60].

In this study, across all professional groups, feedback and rewards were rated the lowest among aspects of transformational leadership. Several previous studies have found similar results in the Finnish healthcare context [19, 45, 52]. However, there has been some improvement over the past 10 years, attributed to the systematic development of nursing leadership in Finland towards a magnet culture [57], which can be considered as an investment in nursing leadership resources [30].

A recent study has found that providing feedback, autonomy, and supporting professional development increases nurses' experience of work engagement and intrinsic motivation [37]. There is a relationship between nurses' job satisfaction and commitment and feedback and rewards. In addition to financial rewards, nonfinancial rewards have proven to be significant. These include appreciation and feedback from the work community; worktime arrangement; work-life balance; access to training and professional development; and opportunity to develop, influence, and participate; feedback; and acknowledgment from managers. These aspects are all considered rewarding and help create a positive and motivating work environment [61, 62]. Nurses seek feedback on their work as it enables their professional development. Managers need to be fair and consider employees individually when giving feedback and rewards. A systematic and fair approach to feedback and recognition fosters a climate of trust, which, in turn, increases work engagement and reduces burnout. Trust between the manager and nurses grows when the manager is present in the workplace, delegates responsibility, and genuinely involves nurses in decision-making. These factors are considered rewarding and enhance nurses' work engagement [28].

In this study, nurses reported a moderate level of work engagement, with the highest scores observed in the dedication dimension. Meta-analyses by Mazzetti et al. [3] have demonstrated that factors such as social support, job control and autonomy, task variety, and feedback are positively associated with work engagement. Similarly, previous research has identified a variety of internal and external factors that influence nurses' commitment to their work [13, 60, 63, 64]. [13] Notably, clinical leadership has been recognized as a significant contributor to enhancing both nurses' work engagement and the quality of care [64]. Work engagement itself plays a critical role in determining the quality of care in healthcare settings. Furthermore, personal characteristics, such as empathy, and positive psychological traits, including self-efficacy, optimism, hope, and resilience, have been positively linked to higher levels of work engagement [13, 63]. Research has shown that commitment across all age groups is influenced by a variety of factors, although gender-related differences have also been observed [5, 65]. Younger generations are driven by evolving work values, skill development, workplace well-being, and community connections. In contrast, older nurses, particularly those from Generation X, tend to prioritize dedication and are primarily motivated by meaningful work and intrinsic rewards. However, the present study found no significant differences in work engagement across different age groups.

This study showed that nurses' assessments of the quality of working unit operations varied across different dimensions of work engagement. The higher nurses assessed their work engagement, the higher they assessed the quality of working unit operations. This pattern was observed across all dimensions of work engagement. The quality of care has become a significant attraction factor in hospital settings [56]. Motivated and engaged nurses are also shown to be satisfied with their work [17, 28]. Schaufeli and Bakker [30] highlight that aspects of vigor and dedication in work engagement represent the positive opposites of exhaustion and cynicism. According to the JD-R theory, transformational leaders provide resources and create an environment that helps employees manage job demands, thus enhancing engagement and performance [46].

Nurses' well-being should also be considered from a patient safety perspective, as work burnout has been found to reduce the quality of nursing care, worsen patient outcomes, decrease work engagement, and increase adverse events such as medication errors and infections [66]. In this study, the higher nurses assessed the quality of work unit operations, the better they assessed their nurse managers' transformational leadership in all aspects. Previous research has shown that a transformational leadership style in nursing is related to the quality of nursing care, leading to better patient outcomes, evidence-based nursing care, nurse job satisfaction, and nurse work commitment [67, 68]. In the light of the JD-R theory [30], particularly the aspect of transformational leadership's support for professional development, which was evaluated at the highest level in this study, it can be stated that transformational leaders support and motivate their staff to provide better quality care [17, 69].

Previous research has noted that transformational leadership is achieved when it is assessed to be at an excellent level [44]. In Kvist et al.'s study [52], transformational leadership among nurse managers did not reach an excellent level. The results of this study align with these findings, indicating that transformational leadership among nurse managers is generally effective but falls short of excellence across all its components. There were differences in nurses' assessments of their managers' transformational leadership between different operational units. Only the mental health and substance abuse services unit reached an excellent level of transformational leadership. This unit achieved a high level of transformational leadership across all components except for providing feedback and rewards, where evaluations remained just under an excellent level. Eneh et al. [50] obtained similar results, showing that nurses working in mental health and substance abuse services perceived transformational leadership, especially in the ethical leadership component, as being better than nurses in other specialty areas. Interestingly, there are still differences between units. One reason for these results could be that the status of the unit influences the nurse managers' own perception of transformational leadership [70]. In the context of mental health, the staff and leaders are often more trained to meet people and interact because of the nature of patient work. This is also reflected in a lower hierarchy between leaders and staff, as well as among different professional groups.

In this study, we explored nurse managers' transformational leadership style and its relationship with nurses' engagement. The JD-R theory supports our findings. Schaufeli and Bakker [30] defined work engagement as comprising vigor, dedication to work, and absorption in work tasks [30]. In essence, an employee experiencing work engagement becomes absorbed in their work, finds it meaningful, and is a motivated worker who genuinely enjoys their job [29–33]. According to the JD-R theory, work engagement improves when job demands and resources are balanced. Transformational leadership promotes work engagement by providing the necessary support and motivation. Essentially, transformational leadership enhances job resources, counterbalancing job demands and fostering a high level of work engagement, which leads to better performance and well-being.

## 6. Limitations

The external validity of the study is limited due to a low response rate (18%), which raises concerns about nonresponse bias. Furthermore, the data were collected from a single hospital in Finland, which may limit the generalizability of the findings to other healthcare settings or countries. As the study reflects the Finnish healthcare context, further research involving larger, more diverse samples across multiple institutions and regions is needed to validate and extend these findings. According to the National Institute for Health and Welfare [71] and WHO [55], on average, the age and gender distribution of the respondents, as well as the professional groups, were representative of the general nursing population. Thus, in terms of demographic representation, the sample reasonably resembled the population. Therefore, the study's findings can be considered insightful for developing nursing management and enhancing nurses' work engagement [53].

The study's measures have been widely used and found to be reliable. The TLS scale, specifically, has been employed in previous nursing studies and has proven to be both reliable and valid. Developed and applied in the context of nursing leadership in Finland [44, 45, 50], the scale is available in Finnish, thereby ensuring that translation does not pose a threat to its validity [53]. The original TLS includes categories assessing both nurse managers' and nurse directors' leadership practices. However, this study focuses solely on the category related to nurse managers' leadership practices, excluding the nurse director category, as they do not directly oversee nursing processes or provide feedback to nurses. Previous studies have reported high reliability for the instrument, with Cronbach's alpha values ranging from 0.878 to 0.97 [45, 50, 52]. In this study, the internal consistency of the measures was also assessed using Cronbach's alpha coefficients, which ranged between 0.82 and 0.97, indicating good reliability.

The UWES-9 scale has been extensively studied internationally and found valid and reliable [3, 29, 30, 32, 33]. The scale has been translated into Finnish by researchers at the Finnish Institute of Occupational Health through international collaboration, and it has been utilized with a Finnish-speaking population [32, 53].

## 7. Conclusion

Transformational leadership by nurse managers can significantly enhance work engagement. Nurse managers play a crucial role in fostering nurse work engagement, which, in turn, is essential for delivering high-quality nursing care. Nurses' well-being impacts not only their ability to cope at work and in their personal lives but also the quality of care patients receive. Through transformational leadership, competent and innovative nurse managers can enhance work engagement, creating a healthy and motivated work atmosphere. The tools of transformational leadership equip nurse managers to support nurses' well-being and work engagement, fostering a climate of trust where everyone can contribute to the development of both individual and collective engagement in the workplace.

In the future, nurse managers should focus more on nurses' well-being and work engagement. Nurses expect to receive more feedback and recognition from their managers. For example, nurse managers can incorporate and develop transformational leadership in their practices through training. More research is needed on the relationship between nursing leaders' leadership style and nurses' well-being to enable nurse managers to systematically improve nurses' well-being. In addition, further research could explore other factors that influence work engagement, as the current predictive power is not high.

## Figures and Tables

**Table 1:** Overall means of the work engagement (UWES-9) and transformational leadership (TLS) subscales, and the relationship between background variables and UWES-9 and TLS subscales.

	*n*	(UWES-9) Vigor *M* (SD)	(UWES-9) Dedication *M* (SD)	(UWES-9) Absorption *M* (SD)	UWES-9 Overall *M* (SD)	(TLS) Ethical leadership *M* (SD)	(TLS) Management of the nursing processes *M* (SD)	(TLS) Giving feedback and rewards *M* (SD)	(TLS) Support for professional development *M* (SD)	(TLS) Overall *M* (SD)
Overall means	154	3.68 (1.19)	4.14 (1.22)	3.80 (1.17)	3.87 (1.12)	3.23 (1.15)	3.25 (0.92)	2.64 (1.02)	3.17 (1.11)	3.15 (0.99)
Position	154									
Registered nurse	129	3.67 (1.20)	4.13 (1.22)	3.82 (1.18)	3.86 (1.12)	3.28 (1.16)	3.26 (0.95)	2.69 (1.00)	3.24 (1.10)	3.19 (1.00)
Paramedic	14	3.71 (1.25)	4.07 (1.26)	3.67 (1.18)	3.82 (1.18)	2.74 (0.79)	3.00 (0.63)	2.06 (0.88)	2.57 (0.86)	2.71 (0.67)
Others	11	3.67 (1.19)	4.39 (1.16)	4.03 (1.22)	4.03 (1.16)	2.49 (1.28)	3.06 (0.93)	2.20 (1.27)	2.37 (1.37)	2.64 (1.12)
*p* value		0.994	0.735	0.604	0.790	0.107	0.409	0.061	0.064	0.146

Age range, years	143									
30 years or under	29	3.69 (1.03)	4.20 (1.06)	3.75 (1.16)	3.88 (0.99)	3.17 (1.03)	3.24 (0.69)	2.52 (0.76)	3.00 (0.91)	3.08 (0.79)
31–40 years	38	3.51 (1.31)	3.95 (1.29)	3.82 (1.15)	3.76 (1.15)	3.27 (1.26)	3.26 (1.02)	2.63 (1.12)	3.28 (1.28)	3.18 (1.12)
41–50 years	34	3.79 (1.08)	4.32 (1.17)	3.89 (1.18)	4.00 (1.10)	3.19 (1.05)	3.12 (0.91)	2.64 (0.95)	3.21 (1.01)	3.09 (0.92)
Over 50 years	42	3.86 (1.07)	4.31 (1.08)	3.99 (0.99)	4.05 (0.99)	3.24 (1.20)	3.34 (0.95)	2.69 (1.11)	3.15 (1.07)	3.19 (1.01)
*p* value		0.743	0.518	0.806	0.692	0.955	0.815	0.968	0.771	0.941

Gender	152									
Woman	134	3.69 (1.14)	4.14 (1.14)	3.85 (1.12)	3.90 (1.06)	3.15 (1.13)	3.18 (0.90)	2.55 (0.98)	3.09 (1.07)	3.07 (0.95)
Man	11	3.15 (1.31)	3.81 (1.52)	3.09 (1.18)	3.35 (1.30)	3.67 (1.12)	3.71 (0.75)	3.21 (1.07)	3.55 (1.03)	3.60 (0.93)
Do not want to say	7	4.0 (1.92)	4.24 (2.03)	3.67 (1.92)	3.97 (1.91)	3.76 (1.44)	3.67 (1.46)	3.12 (1.35)	3.73 (1.65)	3.63 (1.45)
*p* value		0.189	0.544	0.161	0.310	0.150	0.090	0.057	0.123	0.077

Overall work experience in this hospital, years	143									
10 years or under	54	3.58 (1.26)	4.20 (1.18)	3.81 (1.24)	3.86 (1.12)	3.31 (1.15)	3.31 (0.86)	2.66 (1.00)	3.19 (1.14)	3.20 (0.98)
11–20 years	48	3.69 (1.11)	3.99 (1.13)	3.69 (1.07)	3.79 (1.05)	3.15 (1.11)	3.17 (0.95)	2.64 (0.98)	3.20 (1.09)	3.09 (0.98)
Over 20 years	41	3.93 (1.11)	4.37 (1.20)	4.10 (1.06)	4.13 (1.07)	3.28 (1.12)	3.34 (0.91)	2.64 (1.09)	3.19 (1.06)	3.20 (0.96)
*p* value		0.344	0.183	0.188	0.213	0.741	0.702	0.930	0.994	0.825

Work experience in the current unit, years	136									
Under 5 years	60	3.80 (1.07)	4.23 (1.12)	3.94 (1.01)	3.99 (0.97)	3.62 (1.00)	3.49 (0.83)	2.89 (0.97)	3.51 (1.02)	3.45 (0.89)
5–10 years	36	3.70 (1.27)	4.19 (1.16)	3.87 (1.34)	3.92 (1.19)	2.75 (1.20)	2.86 (0.99)	2.40 (1.07)	2.75 (1.14)	2.74 (1.05)
Over 10 years	40	3.57 (1.21)	4.06 (1.27)	3.82 (1.06)	3.81 (1.13)	3.01 (1.07)	3.20 (0.88)	2.41 (0.94)	3.00 (0.99)	3.00 (0.89)
*p* value		0.598	0.812	0.871	0.762	< 0.001	0.004	0.010	0.003	< 0.001

Type of employment	149									
Permanent position	123	3.72 (1.13)	4.15 (1.12)	3.82 (1.14)	3.90 (1.06)	3.21 (1.15)	3.26 (0.91)	2.63 (1.02)	3.16 (1.09)	3.14 (0.98)
Nonpermanent/temporary position	26	3.74 (1.23)	4.42 (1.27)	4.05 (1.03)	4.07 (1.08)	3.32 (1.11)	3.26 (0.90)	2.68 (1.02)	3.24 (1.09)	3.20 (0.98)
*p* value		1.00	0.210	0.571	0.535	0.682	0.914	0.732	0.734	0.774

Working hours	151									
Period/shift work	115	3.74 (1.09)	4.21 (1.11)	3.83 (1.14)	3.93 (1.03)	3.26 (1.08)	3.25 (0.87)	2.64 (1.01)	3.20 (1.09)	3.16 (0.94)
Daytime work	36	3.64 (1.36)	4.13 (1.32)	3.88 (1.10)	3.88 (1.20)	3.23 (1.33)	3.33 (1.04)	2.71 (1.05)	3.21 (1.13)	3.19 (1.10)
*p* value		0.968	0.968	0.774	0.865	0.939	0.668	0.745	0.962	0.827

Operational unit	150									
Acute care and emergency	58	3.61 (1.20)	3.97 (1.23)	3.63 (1.23)	3.74 (1.14)	3.07 (1.00)	3.03 (0.89)	2.46 (0.96)	3.04 (1.05)	2.97 (0.90)
Operational	33	3.67 (1.19)	4.20 (1.16)	3.79 (1.24)	3.89 (1.15)	3.02 (1.19)	3.33 (0.91)	2.53 (1.06)	3.02 (1.22)	3.07 (1.02)
Inpatient wards	21	3.83 (0.86)	4.33 (1.08)	4.14 (0.92)	4.10 (0.88)	3.93 (0.89)	3.60 (0.76)	3.10 (0.79)	3.71 (0.73)	3.66 (0.75)
Outpatient clinics	22	3.56 (1.45)	4.09 (1.40)	3.76 (1.29)	3.80 (1.29)	2.87 (1.26)	2.91 (0.97)	2.30 (0.94)	2.76 (1.05)	2.79 1.02
Women and children's competence center	9	3.96 (1.10)	4.5 (6.76)	4.22 (0.93)	4.25 (0.91)	3.06 (1.31)	3.39 (0.93)	2.80 (1.29)	3.06 (1.39)	3.14 (1.15)
Mental health and substance abuse services	7	4.19 (0.60)	4.76 (0.74)	4.38 (0.56)	4.44 (0.55)	4.61 (0.53)	4.28 (0.60)	3.88 (0.76)	4.43 0.61	4.36 (0.57)
*p* value		0.777	0.434	0.266	0.443	< 0.001	0.002	0.002	0.002	< 0.001

Assessment of the quality of the working unit's operations (scale 0–10)	151									
0–6	33	2.73 (1.19)	3.23 (1.34)	3.09 (1.15)	3.02 (1.15)	2.75 (1.04)	2.70 (0.82)	2.16 (0.89)	2.69 (1.07)	2.64 (0.86)
7–8	77	3.64 (1.01)	4.10 (1.01)	3.73 (1.07)	3.82 (0.94)	3.23 (1.09)	3.28 (0.83)	2.63 (0.96)	3.17 (1.05)	3.15 (0.91)
9–10	41	4.59 (0.70)	5.00 (0.84)	4.60 (0.82)	4.73 (0.73)	3.63 (1.20)	3.63 (0.98)	3.03 (1.06)	3.57 (1.07)	3.54 (1.03)
*p* value		< 0.001	< 0.001	< 0.001	< 0.001	0.004	< 0.001	0.002	0.003	< 0.001

*Note:* UWES-9 Likert scale 0–6 (0 = never, 1 = almost never, 2 = rarely, 3 = sometimes, 4 = often, 5 = very often, and 6 = always). TLS Likert scale (1 = fully disagree, 2 = partially disagree, 3 = I do not know, 4 = partially agree, and 5 = fully agree); *n* = number of respondents; M = mean. Statistical methods: Mann–Whitney *U*-test, Kruskal–Wallis test, Kolmogorov–Smirnov test, t-test, and one-way analysis of variance (ANOVA). The threshold for statistical significance was set at *p* < 0.05.

Abbreviation: SD = standard deviation.

**Table 2:** Correlation matrix between work engagement (UWES-9) and transformational leadership (TLS).

	Ethical leadership	Management of the nursing processes	Giving feedback and rewards	Support for professional development	TLS overall
Vigor	0.387^∗∗^	0.370^∗∗^	0.349^∗∗^	0.357^∗∗^	0.391^∗∗^
Dedication	0.334^∗∗^	0.338^∗∗^	0.337^∗∗^	0.323^∗∗^	0.352^∗∗^
Absorption	0.274^∗∗^	0.293^∗∗^	0.255^∗∗^	0.268^∗∗^	0.293^∗∗^
UWES-9 overall	0.358^∗∗^	0.355^∗∗^	0.344^∗∗^	0.366^∗∗^	0.367^∗∗^

*Note:* Statistical significance: ^∗∗^*p* < 0.01; ^∗^*p* < 0.05.

**Table 3:** Relationships between transformational leadership (TFL) and nurses work engagement (vigor, dedication, and absorption) (*n* = 154).

	*B*	Coefficients			
		Standard error	Beta	*t*	*p* value
TFL/vigor	0.473	0.090	0.391	5.242	< 0.001
*R* ^2^ square	0.153				
*F*	27.478 (*p* < 0.001)				
Standard error of the estimate	1.100				

TFL/dedication	0.435	0.094	0.352	8.987	< 0.001
*R* ^2^ square	0.124				
*F*	21.556 (*p* < 0.001)				
Standard error of the estimate	1.142				

TFL/absorption	0.348	0.092	0.293	8.874	< 0.001
*R* ^2^ square	0.086				
*F*	14.227 (*p* < 0.001)				
Standard error of the estimate	1.127				

*Note: t* = *t*-test; *F* = *F*-test; *B* = regression coefficient; *R*^2^ square = proportion of the variance in the dependent variable.

Abbreviation: TFL = transformational leadership.

## Data Availability

The data that support the findings of this study are available from the corresponding author upon reasonable request.
